# Adipose Tissue-Specific Deletion of 12/15-Lipoxygenase Protects Mice from the Consequences of a High-Fat Diet

**DOI:** 10.1155/2012/851798

**Published:** 2012-12-27

**Authors:** Banumathi K. Cole, Margaret A. Morris, Wojciech J. Grzesik, Kendall A. Leone, Jerry L. Nadler

**Affiliations:** Department of Internal Medicine, Strelitz Diabetes Center, Eastern Virginia Medical School, Norfolk, VA 23507, USA

## Abstract

Type 2 diabetes is associated with obesity, insulin resistance, and inflammation in adipose tissue. 12/15-Lipoxygenase (12/15-LO) generates proinflammatory lipid mediators, which induce inflammation in adipose tissue. Therefore we investigated the role of 12/15-LO activity in mouse white adipose tissue in promoting obesity-induced local and systemic inflammatory consequences. We generated a mouse model for fat-specific deletion of 12/15-LO, *aP2-Cre*; *12/15-LO* ^*loxP/loxP*^, which we call ad-12/15-LO mice, and placed wild-type controls and ad-12/15-LO mice on a high-fat diet for 16 weeks and examined obesity-induced inflammation and insulin resistance. High-fat diet-fed ad-12/15-LO exhibited improved fasting glucose levels and glucose metabolism, and epididymal adipose tissue from these mice exhibited reduced inflammation and macrophage infiltration compared to wild-type mice. Furthermore, fat-specific deletion of 12/15-LO led to decreased peripheral pancreatic islet inflammation with enlarged pancreatic islets when mice were fed the high-fat diet compared to wild-type mice. These results suggest an interesting crosstalk between 12/15-LO expression in adipose tissue and inflammation in pancreatic islets. Therefore, deletion of 12/15-LO in adipose tissue can offer local and systemic protection from obesity-induced consequences, and blocking 12/15-LO activity in adipose tissue may be a novel therapeutic target in the treatment of type 2 diabetes.

## 1. Introduction

Obesity is a rising worldwide epidemic affecting approximately one-third of adults [1]. Obesity is characterized by a persistent exposure to excess nutrients that leads to visceral adiposity and hyperlipidemia, promoting chronic inflammation, insulin resistance, and endoplasmic reticulum stress [2, 3]. This promotes the development of adipocyte dysfunction and systemic decline, including damage to the pancreas, liver, and vascular tissue. Therefore obesity predisposes individuals to the development of type 2 diabetes and cardiovascular disease.

 Increasing evidence from our lab suggests that the 12/15-lipoxygenase (12/15-LO) enzyme plays a critical role in promoting adipocyte dysfunction. 12/15-LO oxygenates polyunsaturated fatty acids to generate many proinflammatory lipid mediators (reviewed in [4]). 12/15-LO is largely involved in metabolizing arachidonic acid to form 12-hydroperoxyeicosatetraenoic acid (12-HPETE) and linoleic acid to form 13-hydroperoxyoctadecadienoic acid (13-HPODE), which are further oxidized to 12-hydroxyeicosatetraenoic acid (12(S)-HETE) and 13-hydroxyoctadecadienoic acid (13-HODE), respectively. Our laboratory has demonstrated that 12/15-LO is activated in white adipocytes from mice fed a high-fat diet and from insulin-resistant Zucker obese rats *in vivo* and is also highly expressed in visceral fat of obese human patients [5–7]. Furthermore, addition of 12/15-LO products to 3T3-L1 adipocytes *in vitro* induces inflammation, insulin resistance, and endoplasmic reticulum stress [5, 6, 8]. Finally, global deletion of 12/15-LO in C57BL/6 mice reduces inflammation and endoplasmic reticulum stress in adipose tissue and preserves insulin sensitivity and pancreatic *β*-cell function when fed a high-fat diet [8–10].

Given that inflammation of adipose tissue is thought to be a preceding step to peripheral metabolic dysfunction [11] and 12/15-LO activity increases inflammation in adipose tissue, we sought to determine whether specific deletion of 12/15-LO in mouse white adipose tissue could protect mice from the local and systemic consequences of a high-fat diet. To this end, we crossed *12/15*-*LO* ^*loxP*/*loxP*^  mice to mice harboring the *Cre* transgene driven by the adipocyte-specific *aP2* promoter (*aP2-Cre*) to generate adipose tissue-specific deletion of 12/15-LO. The adipocyte lipid-binding protein (aP2) is thought to function by facilitating intracellular trafficking of lipids [12]. In addition to adipocytes, aP2 is expressed in macrophages and dendritic cells; however, it is expressed in adipocytes approximately 10,000-fold greater [12]. Furthermore, the use of aP2-Cre mice reveals no recombination of loxP targets in macrophages [13]. Wild type and ad-12/15-LO mice were fed a 60% high-fat diet for 16 weeks, and adipose tissue and systemic organ health were assessed. Our results suggest that a high-fat diet activates 12/15-LO in adipose tissue to promote local and systemic inflammation and that deletion of 12/15-LO in adipose tissue can offer systemic protection from the inflammatory consequences of a high-fat diet.

## 2. Materials and Methods

### 2.1. Animals and Treatments

*12/15*-*LO* ^*loxP*/+^  and *aP2-Cre; 12/15*-*LO* ^*loxP*/+^  mice were generated by Ozgene (Bentley DC, WA, Australia) on a C57BL/6J background. Male C57BL/6J mice were purchased from Jackson Laboratory (Bar Harbor, ME, USA). Mice were housed in a pathogen-free facility at Eastern Virginia Medical School (EVMS), and all experiments were performed in accordance with an animal study protocol approved by the EVMS Institutional Animal Care and Use Committee. Mice were bred to generate homozygous *12/15*-*LO* ^*loxP*/*loxP*^  and *aP2-Cre; 12/15*-*LO* ^*loxP*/*loxP*^  mice. Male mice were placed on a normal chow or high-fat diet (D12331- consisting of 58% of calories from fat, 16.4% of calories from protein, and 25.5% of calories from carbohydrate, primarily sucrose; Research Diets, New Brunswick, NJ, USA) beginning at 8 weeks of age to 16 weeks (*n* = 3 mice per chow diet group and 5-6 mice per high-fat diet group). Blood glucose was measured using OneTouch Ultra2 Glucose Monitors (LifeScan, Milpitas, CA, USA) from tail vein blood samples. Body weight was measured by placing the mice on a scale using the same scale for each set of measurements. At the end of the 16-week diet regimen, mice were euthanized by CO_2_ asphyxiation.

### 2.2. Glucose Tolerance Test (GTT) and Insulin Tolerance Test (ITT)

GTT and ITT were performed as described in [9]. For GTT, mice were fasted overnight and then injected intraperitoneally (IP) with glucose (2 g/kg) in the morning. Blood glucose measurements were performed on tail vein blood samples taken at baseline and at 10, 30, 60, 90, and 120 minutes after injection. The ITT was performed in random-fed mice by IP injection of insulin (0.75 U/kg) in 0.9% NaCl in the afternoon. Blood glucose measurements were performed on tail vein blood samples taken at baseline and at 15, 30, 45, and 60 minutes after injection. 

### 2.3. Serum Measurements

Random-fed mice after the 16-week feeding regimen were euthanized, and blood samples were obtained by cardiac puncture and centrifuged to isolate serum. Insulin and high-molecular-weight adiponectin were measured in serum by ELISA (Mercodia, Uppsala, Sweden and ALPCO Diagnostics, Salem, NH, USA, resp.). 

### 2.4. Adipocyte and Islet Morphometric and Immunohistochemical Analysis

Epididymal adipose and pancreata tissues were isolated and fixed with 10% formalin or Z-fix (Anatech LTD., Battle Creek, MI, USA), respectively, for 4–6 hours at room temperature and embedded in paraffin. 7 *μ*m thick paraffin-embedded tissue sections were then deparaffinized and rehydrated in graduated alcohol in distilled water for processing.

 For morphometric analysis of epididymal adipose tissue, sections were counterstained with hematoxylin and eosin and six representative images per section were collected. Adipocyte area was quantified using ImageJ (NIH website: http://rsb.info.nih.gov/ij/download.html). For immunohistochemical analysis of epididymal fat tissue, sections were subjected to antigen retrieval and stained for Mac2 (Cedarlane, Burlington, NC, USA). Detection was performed with the avidin-biotin-peroxidase method and developed with a diaminobenzidine substrate kit (Vector Laboratories, Inc., Burlingame, CA, USA).

 For morphometric and immunohistochemical analysis of pancreata tissue, sections were stained for insulin (Abcam, Cambridge, MA, USA) and then with Cy-3-conjugated goat anti-guinea pig (Jackson Immunoresearch Laboratories, West Grove, PA, USA) antibodies. Images of all insulin-stained islets from each section (four sections per mouse) were acquired and measured for islet area, percentage of islet area to pancreas area, and percentage of insulin-secreting *β*-cell area per islet area. Islet area and insulin staining were quantified using AxioVision Rel. 4.7 (Carl Zeiss, Oberkochen, Germany).

 Two-dimensional images were acquired using a Zeiss Plan-Apochromat 10X, 0.45 numerical aperture dry objective lens or a 20X, 0.8 numerical aperture dry objective lens on a Zeiss Axio Observer.Z1 microscope with AxioVision Rel. 4.7 software, and a Zeiss AxioCamMRm (fluorescence) or AxioCamHRc (color) camera (Carl Zeiss). All final images were prepared with Adobe Photoshop CS3 Extended, version 10.0.1.

### 2.5. Isolation of White Epididymal Adipocytes and Stromal Vascular Cells (SVCs)

Isolation of white epididymal adipocytes was based on a protocol previously described [14]. Epididymal fat pads were removed from euthanized mice and minced into fine pieces in Krebs Ringer Hepes-BSA (KRHB) buffer (3 mmol/L glucose, 20 nmol/L adenosine, and 10 mg/mL bovine serum) with 1 mg/mL collagenase. Collagenase digestion was performed at 37°C for 1 hour in a shaking water bath. Once digestion of adipose tissue was complete, the cell suspension was filtered through a 0.4 mm Nitex nylon mesh (Sefar America Inc., Kansas City, MO, USA) and washed several times in KRHB buffer, and the adipocytes were allowed to float and processed for RNA or protein extraction. SVCs were then pelleted from the infranatant and processed for RNA extraction.

### 2.6. Islet Isolation

Mouse pancreatic islets were isolated following euthanization as described previously [9, 15]. Briefly, the pancreas was perfused through the common bile duct with 5 mL of 1.4 mg/mL collagenase P and then removed and incubated at 37°C for 8–11 minutes in 1 mL Hank's buffered salt solution. Following incubation, pancreatic tissue was centrifuged, resuspended in Histopaque 1077 (Sigma-Aldrich, St. Louis, MO, USA), and centrifuged again to separate islets from acinar tissue. Islets were then processed for RNA extraction.

### 2.7. RNA Extraction and Real-Time RT-PCR

RNA from adipose tissue or isolated adipocytes was prepared using the RiboPure kit (Ambion, Austin, TX, USA), and RNA from SVCs and islets was prepared using the RNeasy kit (Qiagen, Valencia, CA, USA), according to manufacturer's instructions. cDNA was made from 5 *μ*g of total RNA using MMLV reverse transcriptase (Invitrogen, Carlsbad, CA) in 20 *μ*L reaction volume using random hexamers (Invitrogen). Primer oligonucleotides (see Table 1) with SYBR Green I (Molecular Probes, Carlsbad, CA, USA) or Taqman probes (actin, IL-12p40, TNF*α*, IFN*γ*, IL-1*β*, CX3CL1, IL-10, PPAR*γ*, Adfp, Fasn, DGAT1, and DGAT2; Applied Biosystems, Carlsbad, CA, USA) were used for PCR. All thermal cycling was performed using the CFX96 Thermal Cycler (Bio-Rad, Hercules, CA, USA). All reactions were performed in triplicate, and the data was normalized to the actin housekeeping gene and evaluated using the 2^−ΔΔCT^ method. Expression levels are presented as fold induction/downregulation of transcripts of respective genes relative to control. 

### 2.8. Protein Extraction and Western Blot Analysis

Adipocytes were harvested in RIPA buffer containing protease and phosphatase inhibitors, and equal quantities of protein were separated by SDS-PAGE, transferred to PVDF membrane, and probed at 4°C overnight in either collagen 6 (Abcam) or cleaved caspase-3 (Cell Signaling, Danvers, MA, USA) primary antibody. Detection was performed with secondary HRP-conjugated antibodies and ECL plus (GE Healthcare, Piscataway, NJ, USA) according to manufacturer's instructions. Western blot quantitation was performed by measuring protein band intensities using ImageJ. Protein expression levels were normalized to actin and presented as fold induction/downregulation of band intensities relative to control. All samples represented in the figures and quantified were run on the same gel.

### 2.9. Statistical Analysis

Data are presented as the means ± SEM. Student's *t*-test was used to establish statistically significant differences between samples. A *P* value of <0.05 was considered to indicate statistically significant differences.

## 3. Results

### 3.1. Generation of Adipose Tissue-Specific Deletion of 12/15-Lipoxygenase

**Figure 1:**
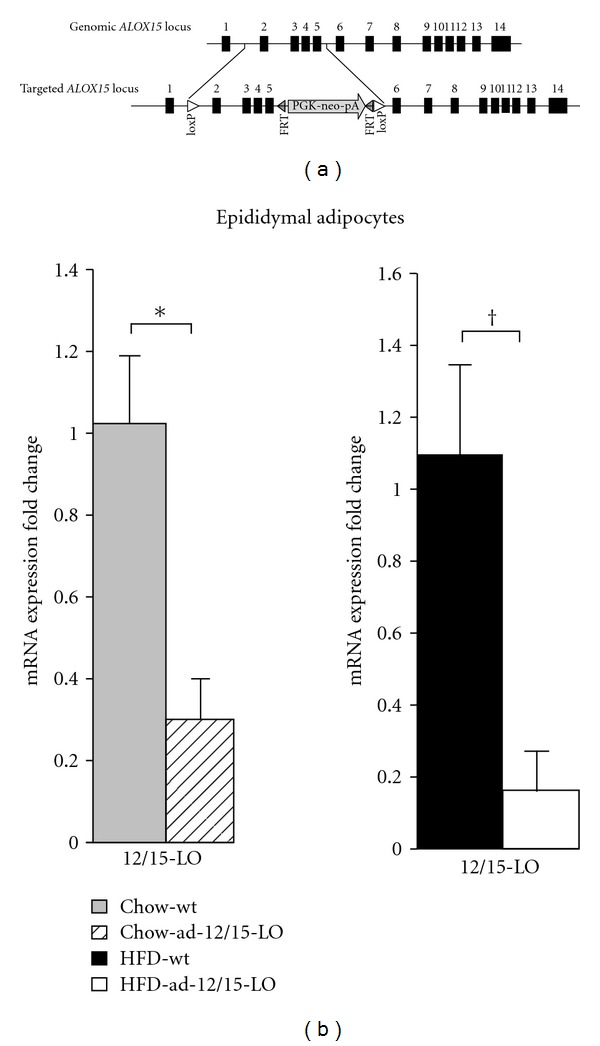
The generation of a targeted conditional knockout allele for the mouse 12/15-lipoxygenase gene, *ALOX15*. (a) The targeted *ALOX15* locus contains loxP sites that flank exons 2 and 5 and a neomycin (neo) cassette selection marker flanked by FRT sites inserted in intron 5. The neo cassette is driven by the mouse phosphoglucokinase gene (PGK) promoter and contains a polyadenylation (pA) signal to terminate the neomycin expression. Exons are depicted as blackened boxes, loxP sites as white triangles, and FRT sites as striped triangles. (b) 12/15-LO mRNA expression was measured in isolated epididymal adipocytes by RT-PCR. All data was normalized to total actin, and the fold changes in expression were calculated relative to control. All data represent the means ± SEM; *n* = 3–6. *P < 0.05 and ^†^ *P* < 0.02 versus control. Chow: chow diet; HFD: high-fat diet; wt: wild type *12/15*-*LO* ^*loxP*/*loxP*^. All measurements are performed on 24-week-old mice.

A conditional knockout allele for the mouse 12/15-lipoxygenase gene, *ALOX15*, was generated by Ozgene and contains loxP sites flanking exons 2–5 (Figure 1(a)). In addition, this allele also contains a neo cassette selection marker that is flanked by FRT sites within intron 5. Heterozygous *12/15*-*LO* ^(*neo*)*loxP*/+^ mice on a C57BL/6J background were generated and subsequently crossed to a Flp-deleter mouse line on a C57BL/6J background to facilitate Flp recombinase-mediated removal of the neo cassette. In addition, Ozgene crossed *12/15*-*LO* ^*loxpP*/+^ mice to an aP2-Cre mouse line to generate *aP2-Cre; 12/15*-*LO* ^*loxP*/+^. We have since bred *12/15*-*LO* ^*loxpP*/+^ and *aP2-Cre; 12/15*-*LO* ^*loxP*/+^ mice to homozygosity for the *12/15*-*LO* ^*loxP*^ allele. *aP2-Cre; 12/15*-*LO* ^*loxP*/*loxP*^ mice will be called ad-12/15-LO mice throughout the paper.

 To ensure specific deletion of 12/15-lipoxygenase (12/15-LO) from white adipocytes, we isolated epididymal adipocytes from 24-week-old male wild type *12/15*-*LO* ^*loxP*/*loxP*^ and ad-12/15-LO mice and measured 12/15-LO mRNA expression. 12/15-LO mRNA expression was significantly reduced by approximately 70% in isolated epididymal adipocytes from ad-12/15-LO adipocytes compared to wild type adipocytes (Figure 1(b)). This reduction in 12/15-LO mRNA expression in adipocytes remained significantly low (by approximately 80%) even when age-matched mice were on the high-fat diet for 16 weeks (Figure 1(b)).

### 3.2. ad-12/15-LO Mice Exhibit Improved Glucose Metabolism When on a High-Fat Diet

**Figure 2:**
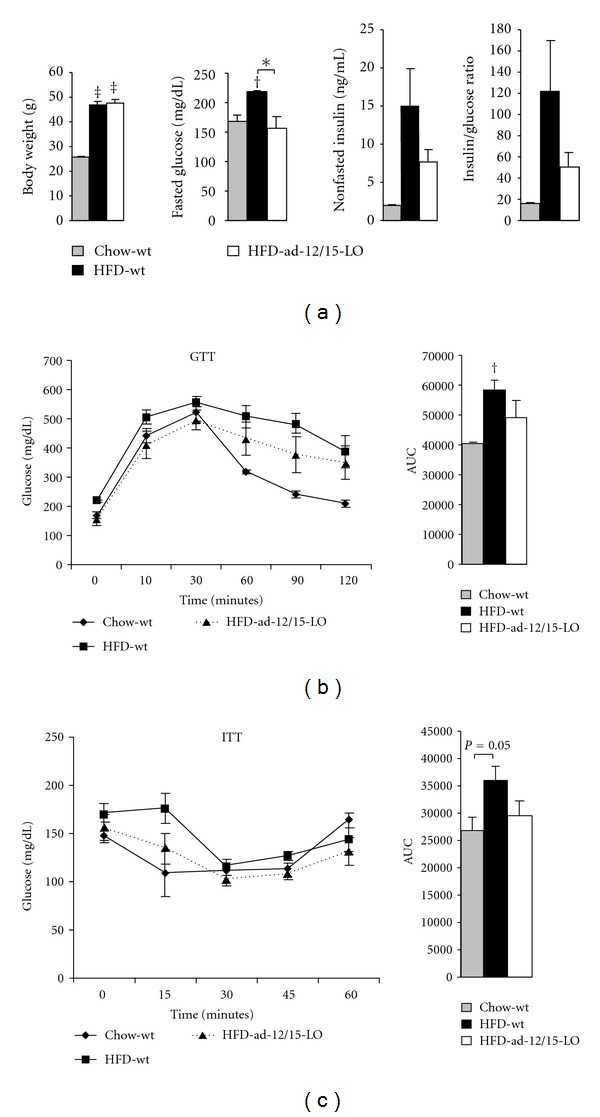
Fat-specific 12/15-LO-deficient mice exhibit improved glucose metabolism when fed a high-fat diet. (a) Body weight, nonfasted serum insulin, and nonfasted serum insulin/nonfasted blood glucose ratio were measured at the end of the 16-week feeding regimen. Fasted blood glucose was measured in overnight fasted mice at 15 weeks of the feeding regimen. Serum insulin levels were measured by ELISA and tail vein blood glucose by glucometers. (b)-(c) Intraperitoneal glucose tolerance test (GTT) and insulin tolerance test (ITT) were performed in mice after 15 weeks of feeding, and blood glucose measurements were measured at the indicated time points. Areas under the curves (AUC) are presented for GTT and ITT. All data represent the means ± SEM; *n* = 3–6. *P < 0.05, ^†^ *P* < 0.02, ^‡^ *P* < 0.001 versus control, unless otherwise indicated. Chow: chow diet; HFD: high-fat diet; wt: wild type *12/15*-*LO* ^*loxP*/*loxP*^.

8-week-old male wild type *12/15*-*LO* ^*loxP*/*loxP*^ control mice (*n* = 5) and ad-12/15-LO mice (*n* = 6) were placed on a high-fat diet for 16 weeks, along with 8-week-old male chow-fed wild type *12/15*-*LO* ^*loxP*/*loxP*^ control mice (*n* = 3), to determine the role of 12/15-LO in adipose tissue in promoting whole-body metabolic dysfunction. At the end of the 16-week feeding regimen, while the high-fat diet-fed groups exhibited significant weight gain compared to chow-fed wild type, no differences in body weight were seen in the high-fat diet-fed wild type and ad-12/15-LO mice (Figure 2(a)). Additionally, fasting blood glucose levels after 15-weeks of feeding were significantly reduced in high-fat diet-fed ad-12/15-LO mice compared to high-fat diet-fed wild type mice and remain unchanged from chow-fed wild type; nonfasting insulin levels after 16-weeks of feeding were also reduced in high-fat diet-fed ad-12/15-LO mice compared to high-fat diet-fed wild type mice (Figure 2(a)). Furthermore, measurement of nonfasting insulin : glucose ratio provides a reading of insulin sensitivity; indeed, this ratio is increased in high-fat diet-fed wild type mice compared to chow-fed wild type mice, and this increase is ameliorated in the high-fat diet-fed ad-12/15-LO mice (Figure 2(a)). Finally, high-fat diet-fed ad-12/15-LO mice exhibited improvements in glucose metabolism compared to high-fat diet-fed wild type mice, as measured by intraperitoneal glucose and insulin tolerance tests (Figures 2(b) and 2(c)).

### 3.3. Adipose Tissue of ad-12/15-LO Mice on a High-Fat Diet Is Less Inflamed

**Figure 3:**
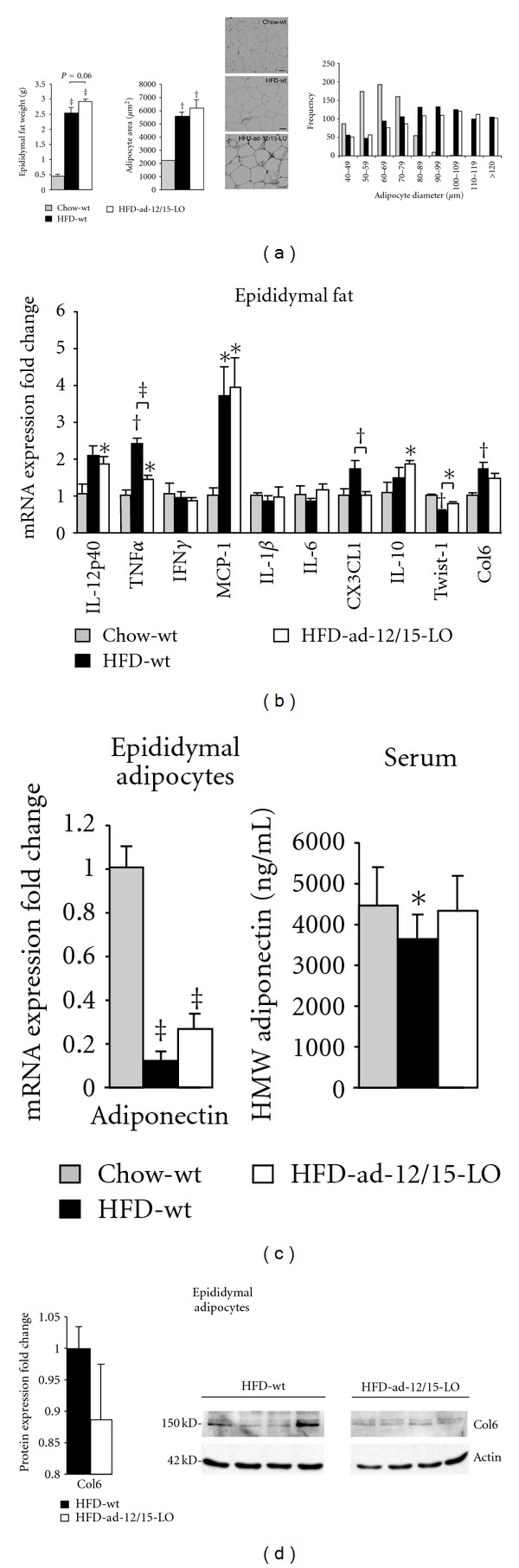
Fat-specific 12/15-LO-deficient mice exhibit decreased inflammation in adipose tissue following a high-fat diet regimen. (a) Epididymal adipose tissue weight and adipocyte size were measured in mice after the 16-week feeding regimen. Hematoxylin and eosin stained sections of epididymal adipose tissue are shown. Scale bar = 50 *μ*M. (b) mRNA measurements of key proinflammatory and anti-inflammatory genes were examined by RT-PCR in epididymal adipose tissue from mice. (c) Adiponectin mRNA and high-molecular-weight (HMW) adiponectin protein were measured in isolated epididymal adipocytes and serum by RT-PCR and ELISA, respectively. (d) Protein measurement of collagen 6 (Col6) was performed from isolated epididymal adipocytes and quantified. Representative western blots are shown, and separate panels for each antibody represent the same exposure from the same gel. All mRNA and protein data were normalized to total actin, and the fold changes in expression were calculated relative to control. All data represent the means ± SEM; *n* = 3–6. *P < 0.05, ^†^ *P* < 0.02, ^‡^ *P* < 0.001 versus control, unless otherwise indicated. Chow: chow diet; HFD: high-fat diet; wt: wild type *12/15*-*LO* ^*loxP*/*loxP*^.

To examine local effects of 12/15-LO deficiency in adipose tissue when mice are on a high-fat diet, we performed morphometric analysis of epididymal adipose tissue from these mice. Mice on a high-fat diet exhibit enlarged fat pads and adipocytes due to increased triglyceride storage of excess fatty acids [2]. While no significant differences were observed between epididymal adipose tissue weight and adipocyte size in wild type and ad-12/15-LO mice on a high-fat diet, there may be a trend for enlargement of adipocyte size in the ad-12/15-LO mice fed a high-fat diet (Figure 3(a)).

Furthermore, we measured mRNA expression of several key proinflammatory genes in epididymal adipose tissue from the chow- and high-fat diet-fed wild type and ad-12/15-LO mice. Chronic inflammation of adipose tissue is accompanied with increased production of cytokines, such as tumor necrosis factor-*α* (TNF*α*) [2]. Indeed, TNF*α* mRNA expression was significantly upregulated in adipose tissue by the high-fat diet in wild type mice; however, fat-specific 12/15-LO deficiency significantly prevented this upregulation (Figure 3(b)). CX3CL1 (or fractalkine), a cytokine derived from the endothelium that promotes atherogenesis, has recently been shown to also be increased in adipose tissue of obese patients and is associated with inflammation, insulin resistance, and type 2 diabetes in these patients [16, 17]. Interestingly, fat-specific 12/15-LO deficiency was also able to significantly prevent the high-fat diet-induced increase in CX3CL1 mRNA expression (Figure 3(b)). 12/15-LO exerts proinflammatory responses in part by upregulating the expression of interleukin-12 (IL-12) in activated inflammatory cells [5, 6, 18–20]. Indeed, a trend for decreased IL-12p40 mRNA expression was observed in the ad-12/15-LO mice fed a high-fat diet compared to wild type mice. The anti-inflammatory cytokine interleukin-10 (IL-10) [21] and Twist-1, a basic helix-loop-helix transcription factor whose expression in human white adipose tissue is inversely correlated with inflammation and insulin resistance [22], are also increased in high-fat diet-fed ad-12/15-LO mice compared to wild type mice (Figure 3(b)). Other proinflammatory cytokines measured revealed no differences between the wild type and ad-12/15-LO mice on a high-fat diet (interferon-*γ* (IFN*γ*), monocyte chemoattractant protein-1 (MCP-1), interleukin-1*β* (IL-1*β*), and interleukin-6 (IL-6); Figure 3(b)).

Adiponectin is an adipose tissue-specific secreted adipokine that is a critical insulin-sensitizer and is downregulated by high-fat diets [23]. Indeed, fat-specific 12/15-LO deficiency ameliorates the significant decrease in adiponectin mRNA expression as seen in isolated epididymal adipocytes from wild type mice on a high-fat diet (Figure 3(c)); a similar effect is observed in serum levels of the active high-molecular-weight form of adiponectin protein (Figure 3(c)) [23].

Furthermore, given the trend for increased adipocyte size in the ad-12/15-LO mice fed a high-fat diet, we examined collagen 6 (Col6). Col 6 is associated with increased fibrosis of inflamed adipose tissue [24]. While Col6 mRNA expression significantly increased in adipose tissue from wild type mice when fed a high-fat diet, fat-specific 12/15-LO deficiency is able to ameliorate this increase (Figure 3(b)); a similar trend was observed for Col6 protein in isolated adipocytes (Figure 3(d)).

### 3.4. Adipose Tissue of ad-12/15-LO Mice on a High-Fat Diet Exhibits Decreased Macrophage Infiltration

**Figure 4:**
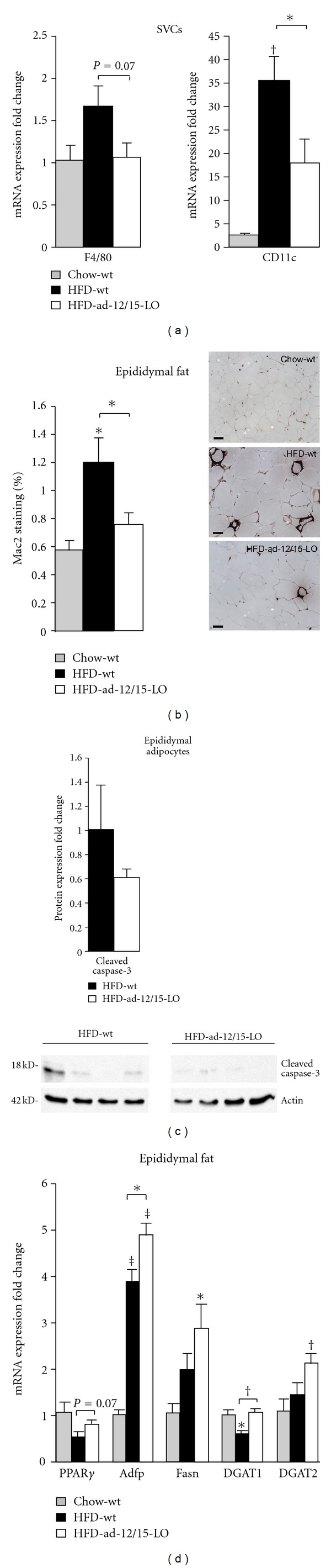
Adipose tissue from fat-specific 12/15-LO-deficient mice exhibits decreased macrophage infiltration and may be more adipogenic and lipogenic. (a) mRNA expression of key macrophage markers was measured by RT-PCR in stromal vascular cells (SVCs) isolated from epididymal adipose tissue. (b) Immunohistochemistry for Mac2 expression was performed in epididymal adipose tissue, and Mac2 staining was quantified. Scale bar = 50 *μ*M. (c) Protein measurements of cleaved caspase-3 were performed from isolated epididymal adipocytes and quantified. Representative western blots are shown, and separate panels for each antibody represent the same exposure from the same gel. (d) mRNA measurements of key adipogenic and lipogenic genes were examined by RT-PCR in epididymal adipose tissue from mice. All mRNA and protein data were normalized to total actin, and the fold changes in expression were calculated relative to control. All data represent the means ± SEM; *n* = 3–6. *P < 0.05, ^†^ *P* < 0.02, ^‡^ *P* < 0.001 versus control, unless otherwise indicated. Chow: chow diet; HFD: high-fat diet; wt: wild type *12/15*-*LO* ^*loxP*/*loxP*^.

To further probe the inflammatory consequences in adipose tissue of fat-specific 12/15-LO deletion when mice are on a high-fat diet, we examined macrophage infiltration into the epididymal fat pad as this is associated with visceral adiposity [25]. Analysis of markers for activated macrophages, F4/80 and CD11c, in isolated macrophage-containing stromal vascular cells of the epididymal adipose fat pad revealed that fat-specific 12/15-LO deletion was able to prevent the increase in F4/80 and CD11c expression seen in wild type mice fed a high-fat diet (Figure 4(a)). Furthermore, immunohistochemical staining of epididymal adipose tissue for the activated macrophage marker, Mac2, revealed that a high-fat diet significantly increased Mac2 staining in wild type mice but not in ad-12/15-LO mice (Figure 4(b)). Finally, a decrease in cleaved caspase-3 protein in isolated adipocytes from high-fat diet-fed ad-12/15-LO mice compared to wild type suggests a decrease in apoptotic adipocytes (Figure 4(c)). Thus it appears that fat-specific 12/15-LO deficiency is able to reduce the local inflammatory consequences in fat normally rendered by a high-fat diet, resulting in decreased macrophage infiltration.

### 3.5. Adipose Tissue of ad-12/15-LO Mice on a High-Fat Diet May Be More Adipogenic and Lipogenic

We further examined expression of genes important in adipocyte differentiation and health. Peroxisome proliferator-activated receptor-*γ* (PPAR*γ*) is a strict requirement for adipocyte differentiation, and formation of new adipocytes generally is thought to indicate the prevalence of healthy adipocytes [26]. Indeed, PPAR*γ* mRNA expression is decreased by a high-fat diet in wild type mice; however, fat-specific 12/15-LO deficiency is able to ameliorate PPAR*γ* decrease when mice are fed a high-fat diet (Figure 4(d)). Additionally, we examined adipose differentiation related protein (Adfp), fatty acid synthase (Fasn), and acyl CoA: diacylglycerol acyltransferases 1 and 2 (DGAT1 and 2) expression. Adfp is a perilipin that plays a role in lipid droplet formation, and stabilization and its expression are associated with increased lipid load, while Fasn is important for the synthesis of long-chain fatty acids [27, 28]. Furthermore, DGAT1 and 2 are important for triglyceride synthesis [29]. Consistent with the observed trend that fat-specific 12/15-LO deficiency may promote larger adipocytes in mice fed a high-fat diet, we observe that Adfp and Fasn mRNA expression is higher in the ad-12/15-LO mice compared to wild type mice when fed a high-fat diet (Figure 4(d)). Also, while DGAT1 mRNA expression is significantly decreased when wild type mice are fed a high-fat diet, its expression remains unchanged in the high-fat diet-fed ad-12/15-LO mice, and DGAT2 mRNA expression is significantly increased in the high-fat diet-fed ad-12/15-LO but not wild type mice (Figure 4(d)). 

### 3.6. /15-LO Deficiency in Fat Alters Serum Lipid Levels

Given the reduced inflammation in adipose tissue in the context of fat-specific 12/15-LO deficiency when mice are fed a high-fat diet, we examined serum levels of lipids that can be highly affected by adipocyte health. In particular, total cholesterol and nonesterified free fatty acids were similarly significantly increased in both wild type and ad-12/15-LO mice fed a high-fat diet; however, large changes in triglycerides were not observed (data not shown).

### 3.7. /15-LO Deficiency in Fat Restores *β*-Cell Function

**Figure 5:**
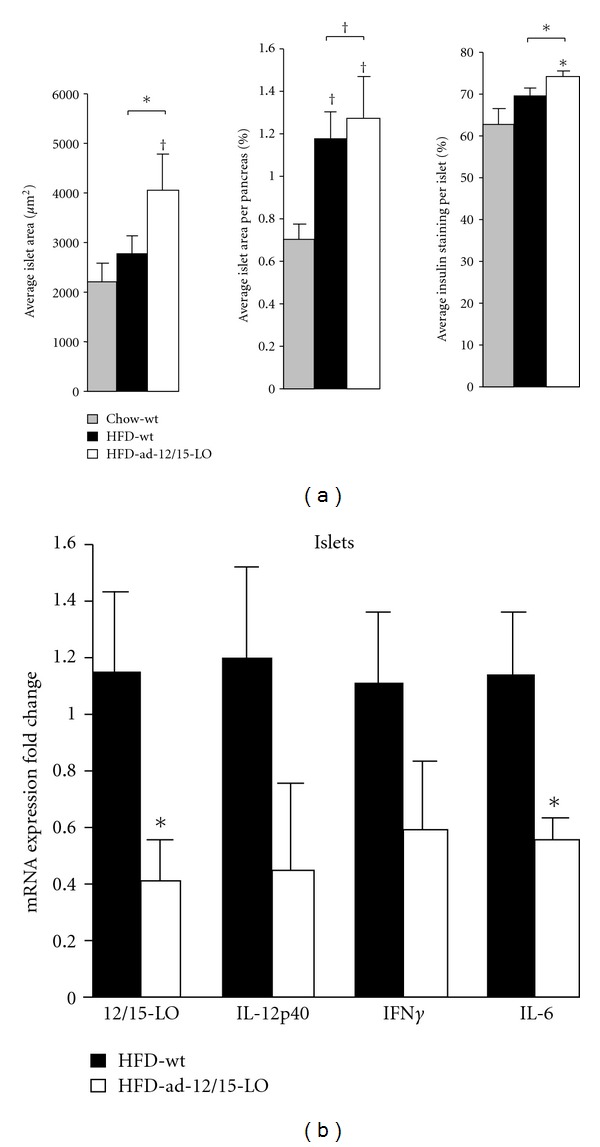
Fat-specific 12/15-LO-deficient mice exhibit improvements in pancreatic islet health. (a) Immunohistochemistry for insulin expression on pancreatic sections from mice after the 16-week feeding regimen was analyzed for average islet area, average islet area per pancreas area, and average insulin staining per islet; *n* = 3–5; Chow: chow diet; HFD: high-fat diet; wt: wild type *12/15*-*LO* ^*loxP*/*loxP*^. (b) mRNA measurements of key proinflammatory genes were examined by RT-PCR in isolated pancreatic islets from mice after the 16-week feeding regimen; *n* = 6–8; HFD: high-fat diet; wt: wild type C57BL/6J. All mRNA data were normalized to total actin, and the fold changes in expression were calculated relative to control. All data represent the means ± SEM. *P < 0.05 and ^†^ *P* < 0.02 versus control, unless otherwise indicated.

Inflammation in adipose tissue is thought to precede the development of inflammation elsewhere and is thus an early indicator for disease development in peripheral tissues [11, 30]. Given that pancreatic *β*-cells are an early key target tissue in obesity-induced type 2 diabetes, we evaluated whether the beneficial effects of fat-specific 12/15-LO deficiency are manifested in the pancreatic islets. C57BL/6 mice fed a high-fat diet are known to undergo islet hyperplasia as a mechanism to meet increased demands for insulin production given the rise in insulin resistance. Interestingly, while high-fat diet-fed wild type mice exhibit a trend for larger pancreatic islets compared to chow-fed wild type mice, the high-fat diet-fed ad-12/15-LO mice display even larger islets (Figure 5(a)); a similar trend is observed for total islet area per pancreas area (Figure 5(a)). In addition, the percentage of insulin-stained *β*-cells per islet slightly increases in the high-fat diet-fed wild type mice compared to chow-fed wild type mice, and this is further augmented in the high-fat diet-fed ad-12/15-LO mice (Figure 5(a)). In a separate study where wild type C57BL/6J and ad-12/15-LO mice were fed a high-fat diet for 16 weeks, islet gene expression was measured for proinflammatory cytokines. 12/15-LO and IL-6 mRNA expression was significantly reduced, and IL-12p40 and INF*γ* mRNA expression was reduced in islets from ad-12/15-LO mice compared to wild type mice fed a high-fat diet (Figure 5(b)). Islets from chow-fed wild type and ad-12/15-LO age-matched control mice were analyzed for 12/15-LO mRNA expression, and no differences were observed (data not shown), suggesting the *aP2-Cre* transgene is specific to fat and the exhibited changes in 12/15-LO mRNA expression in islets from mice on a high-fat diet were a consequence of inflammation. Thus, it appears that deletion of 12/15-LO in adipose tissue is able to confer protection to *β*-cell function.

## 4. Discussion

Obesity is marked by an increased demand for intracellular lipid storage of excess circulating free fatty acids, leading to adipocyte stress and ensuing chronic inflammation and macrophage infiltration [2]. Production of proinflammatory cytokines by adipose tissue further leads to local and systemic insulin resistance. The 12/15-lipoxygenase (12/15-LO) enzyme generates proinflammatory lipids, such as 12(S)-HETE, which promote local dysfunction in various tissues such as adipose, pancreatic, and vascular tissues (reviewed in [4]). We show here that local chronic activation of 12/15-LO in adipose tissue by a high-fat diet is sufficient to drive local adipocyte dysfunction with ensuing systemic decline.

Inflammation in adipose tissue induced by a high-fat diet is characterized by increased expression of proinflammatory cytokines, such as TNF*α*. TNF*α* decreases insulin sensitivity, exacerbates inflammation, and inhibits adipogenesis [2]. TNF*α* is generated predominantly by infiltrating macrophages and promotes increased lipolysis [2, 25]. These in effect lead to increased circulating free fatty acids that promote ectopic lipid accumulation and insulin resistance. Indeed, we observed that ad-12/15-LO mice fed a high-fat diet did not demonstrate increased TNF*α* expression, consistent with improved insulin sensitivity and overall decreased inflammation. Interestingly, low expression of Twist-1 in human adipose tissue is correlated with high levels of TNF*α* expression, consistent with our results, and Twist-1 expression in Th1 lymphocytes limits TNF*α* expression [22, 31]. Furthermore, IL-10 overexpression in mice is able to prevent diet-induced insulin resistance [32], and indeed we see increased IL-10 expression in the ad-12/15-LO mice fed a high-fat diet with concomitant improvements in insulin sensitivity. Thus the absence of chronic 12/15-LO activation in adipose tissue in obese conditions is able to prevent the phenotypic proinflammatory state induced by a high-fat diet.

The complex inflammatory cascade and cell death elicited in adipose tissue by obesity are responsible for the characteristic feature of macrophage infiltration into the fat pad [33]. MCP-1 is thought to be one of the key adipokines in this process. We observed that macrophage infiltration into the epididymal fat pad of high-fat diet-fed ad-12/15-LO mice is significantly reduced compared to the wild type mice in agreement with the decreased TNF*α* expression (likely secreted by the macrophages); however, while MCP-1 mRNA expression was high in the high-fat diet-fed groups, no difference was observed between the groups. This may suggest that another key chemoattractant player is important for eliciting macrophage infiltration given the absence of macrophages in the high MCP-1-expressing ad-12/15-LO fat, a possibility supported by the observation that MCP-1 knockout mice still exhibit obesity-induced macrophage infiltration into adipose tissue [34]. This additional chemoattractant would be dependent upon chronic 12/15-LO activation whereby specific lipid metabolites, such as 12(S)-HETE, may be responsible for eliciting the inflammatory cascade and subsequent macrophage infiltration. Alternatively, the decreased macrophage infiltration could be explained by the role of 12/15-LO to induce endothelial expression of intercellular adhesion molecule-1 (ICAM-1) [35]. ICAM-1 mediates monocyte adhesion to the vascular wall endothelium and is required for transendothelial migration of monocytes into the surrounding tissue. Therefore, the absence of 12/15-LO may simply lead to impaired transendothelial migration and subsequent decreased macrophage infiltration into the adipose tissue. Furthermore, normal recruitment of macrophages to the fat pad occurs when adipocytes undergo lipolysis or cell death and macrophages are activated to uptake the free fatty acids or engulf apoptotic cells to protect local adipocyte and distal tissue health [36, 37]. However, chronic activation of these macrophages in the obese state promotes a phenotypic proinflammatory switch in the macrophages that is detrimental to local adipocyte health and metabolism [36, 37]. Thus in the ad-12/15-LO mice fed a high-fat diet, less macrophages may be present due to decreased lipolysis and cell death.

 Additionally, *de novo* adipogenesis and lipogenesis accompanied by decreased lipolysis are important for insulin sensitivity and longevity as this process promotes the development of healthy, active adipocytes and removes the excess circulating fatty acids to prevent ectopic lipid accumulation and ensue inflammation and insulin resistance in nonadipose tissue [28]. On the contrary, TNF*α* inhibits adipogenesis and lipogenesis in part through downregulating expression of PPAR*γ* and its lipid droplet protein targets, respectively [2, 25]. Our data suggest that adipocytes from ad-12/15-LO mice versus wild type mice fed a high-fat diet may display increased adipogenic and lipogenic potential given the increased expression of PPAR*γ*, Adfp, Fasn, DGAT1, and DGAT2 with a trend for decreased circulating free fatty acids in the serum. This is of importance given the evidence describing a positive correlation between high lipid-droplet protein expression and insulin sensitivity in patient fat samples [38] and studies demonstrating that mice overexpressing DGAT1 in adipocytes and macrophages were protected from diet-induced inflammation and insulin resistance [39]. While not significant, it is possible that there is a tendency for ad-12/15-LO adipocytes to become larger compared to wild type adipocytes after a high-fat feeding. This may suggest a greater tendency for these adipocytes to increase their lipid load, in agreement with the increased expression of lipogenic genes. Interestingly, Col6 is a component of extracellular matrix and Col6-deficient* ob/ob* mice display larger but healthier adipocytes since they can expand in size in response to increased lipid loading without being subjected to shear stress. Indeed, ad-12/15-LO adipocytes seem to exhibit lower levels of Col6. Furthermore, while white adipose tissue mass is greater in the high-fat diet-fed ad-12/15-LO mice compared to the high-fat diet-fed wild type mice, no changes in overall body weight were observed. This may suggest differences in lean versus fat mass and warrants further investigation to determine the role of 12/15-LO in partitioning of lipids in adipose tissue. Finally, while analysis of fatty acid and triglyceride serum levels was not different between the high-fat diet-fed ad-12/15-LO and wild type nonfasted mice, analysis of serum from fasted mice will likely reveal a greater difference than seen in this study and thereby further decipher the role of 12/15-LO in adipogenesis and lipogenesis. This is important as a 16-week high-fat diet is likely overloading the adipocyte, and thus differences in serum levels of fatty acids and triglycerides between the strains are hard to discern in the nonfasted state.

 Adipose tissue has an important role in controlling whole-body glucose homeostasis such that pancreatic islets are highly susceptible to the damaging effects elicited by circulating cytokines and fatty acids released by inflamed adipose tissue [40]. Within the first week of high-fat feeding in mice, insulin resistance primes the islets to respond by becoming hyperplastic to meet the increased demands of insulin production to compensate for insulin resistance and thereby maintain normal blood glucose levels [41]. Furthermore, the islets themselves become inflamed and begin to decline in insulin production and secretion [41]. Indeed, fat-specific 12/15-LO deficiency may protect *β*-cell function as evident by decreased islet expression of proinflammatory genes, such as IL-12p40, IFN*γ*, and IL-6. Furthermore, while it is generally accepted that hyperplasia is an adaptive response to overcome insulin resistance, we observed that the ad-12/15-LO mice exhibited even larger islets despite their improvements in glucose metabolism. The decreased inflammatory environment in these mice may allow this hyperplastic response to ensue without facing the normal detrimental consequences of inflamed islets. This is a novel aspect of the study that will certainly be investigated in future studies as an interesting crosstalk clearly exists between 12/15-LO activity in adipose tissue and pancreatic islet inflammation and *β*-cell function.

 aP2-Cre can exhibit low levels of expression in macrophages and is of concern in the present study. However, the *aP2-Cre* transgene in our mouse model appears to be specific to adipocytes and not leaky in macrophages as thioglycollate-induced peritoneal macrophages isolated from wild type and ad-12/15-LO mice exhibit similar IL-12 expression (data not shown). This is important as 12/15-LO is a requirement for IL-12 expression in macrophages [42]. Thus this further supports the idea that inflamed adipose tissue driven by 12/15-LO has significant systemic impact apart from macrophages. Future studies comparing isolated macrophages from *aP2-Cre; 12/15*-*LO* ^*loxP*/*loxP*^ as well as *adiponectin-Cre; 12/15*-*LO* ^*loxP*/*loxP*^ mice (which we are currently generating) will provide further information on the molecular and functional consequences of aP2-Cre in macrophages as adiponectin is expressed only in adipocytes.

 Recent data suggests that inflammation in adipose tissue precedes the development of disease progression in peripheral tissues [11, 30]. In particular, Stanton et al. reported that inflammatory gene expression changes in epididymal adipose tissue of C57BL/6 mice in response to a high-fat feeding occur as early as 6 weeks of feeding; by 16 weeks of feeding there is a shift in inflammatory changes in the adipose tissue to the liver. These results suggest that analysis of adipose tissue as early as 6 weeks of high-fat feeding may reveal even more dramatic differences in epididymal adipose tissue of wild type versus ad-12/15-LO mice. Furthermore, careful analysis of liver tissue from 6–16 weeks of high-fat feeding will also reveal whether fat-specific 12/15-LO deficiency can protect mice from obesity-induced fatty liver disease.

If indeed controlling obesity-induced dysfunction of adipose tissue metabolism will protect systemic tissues from the inflammatory adipokines secreted by adipose tissue, then one would suspect to observe significant decreases in circulating cytokine levels in the serum. While serum cytokine levels were not evaluated in this study due to lack of material, we would expect serum cytokine levels to be reduced in the high-fat diet-fed ad-12/15-LO mice compared to wild type. These cytokines likely will reflect the inflammatory expression profile in adipose tissue as this organ is much larger than other cytokine-contributing tissues and thus adipose tissue adipokine production may more significantly impact circulating systemic cytokine levels. 

## 5. Conclusions

In summary, we report the first study utilizing a targeted tissue-specific deletion of 12/15-LO. The use of *12/15*-*LO* ^*loxP*/*loxP*^  mice will allow targeted removal of 12/15-LO in various tissues affected by diabetes and cardiovascular disease, including *β*-cells, liver, and vascular tissue. In addition, our findings suggest that 12/15-LO-mediated pathways in adipocytes play a role in obesity-associated insulin resistance by modulating adipose tissue inflammatory pathways. Inactivation of 12/15-LO in fat was able to relieve local inflammation resulting in positive systemic metabolic consequences. Therefore, deciphering the key molecular players regulating 12/15-LO expression in adipose tissue may have therapeutic value in the treatment of obesity-related disorders, such as diabetes and cardiovascular disease.

## Figures and Tables

**Table 1:** Primer sequences used in SYBR-based real-time RT-PCR amplification of mouse cDNA.

Mouse genes	Forward primer (5′–3′)	Reverse primer (5′–3′)
12/15-LO	ctctcaaggcctgttcagga	gtccattgtccccagaacct
Actin	aggtcatcactattggcaacga	cacttcatgatggaattgaatgtagtt
Adiponectin	gttgcaagctctcctgttcc	atccaacctgcacaagttcc
CD11c	ctgagagcccagacgaagaca	tgagctgcccacgataagag
Collagen 6 alpha 1	gatgagggtgaagtgggaga	cagcacgaagaggatgtcaa
F4/80	ctttggctatgggcttccagtc	gcaaggaggacagagtttatcgtg
IL-6	gaggataccactcccaacagacc	aagtgcatcatcgttgttcataca
MCP-1	cttctgggcctgctgttca	ccagcctactcattgggatca
Twist-1	cgcacgcagtcgctgaacg	gacgcggacatggaccagg
